# The influence of socioeconomic status on the decision to use bilateral internal mammary artery grafting in coronary artery bypass surgery

**DOI:** 10.1016/j.xjon.2025.10.021

**Published:** 2025-10-31

**Authors:** Yu Hohri, Tanner Powley, Chunhui Wang, Pengchen Wang, Paul Kurlansky, Koji Takeda

**Affiliations:** Division of Cardiothoracic and Vascular Surgery, Columbia University Medical Center, New York, NY

**Keywords:** coronary artery bypass grafting, bilateral internal mammary artery, arterial revascularization, socioeconomic status, Distressed Communities Index

## Abstract

**Objective:**

The influence of socioeconomic status on the decision to use bilateral internal mammary artery (BIMA) grafting versus single IMA (SIMA) grafting remains uncertain. In this study, we examine the association between Distressed Communities Index scores and the decision to use BIMA grafting.

**Methods:**

This multicenter retrospective study includes patients who underwent primary coronary artery bypass grafting with BIMA or SIMA between 2015 and 2024. Patients with 1 distal anastomosis and without an IMA graft were excluded. The Distressed Communities Index is a validated, zip code-based metric that reflects socioeconomic distress using 7 indicators, with higher scores indicating greater distress. It was used to assess the association between socioeconomic factors and both the likelihood of receiving BIMA grafting and postoperative outcomes.

**Results:**

Of 17,110 patients, 13,692 patients (80.0%) received SIMA grafting, whereas 3418 patients (20.0%) received BIMA grafting. The median age was different between 2 groups (63.0 years; range, 56.0-70.0 years vs 68.0 years; range, 61.0-74.0 years; *P* < .001), and BIMA was more frequently used in patients with fewer comorbidities than SIMA (all *P* values < .05). The median Distressed Communities Index score was 45.80 (range, 24.29-70.63) in BIMA and 44.03 (range, 23.39-68.47) in SIMA grafting (*P* < .001). Multivariable logistic regression revealed that Distressed Communities Index score was associated with the likelihood of receiving BIMA grafting (odds ratio, 0.997; 95% CI, 0.995-0.99; *P* < .001), but not with any postoperative outcomes (all *P* values > .05).

**Conclusions:**

Patients from more distressed communities are less likely to receive BIMA grafting. This suggests that surgeons may unknowingly consider socioeconomic factors as part of their decision making for BIMA grafting.

## Graphical Abstract

**Figure:**
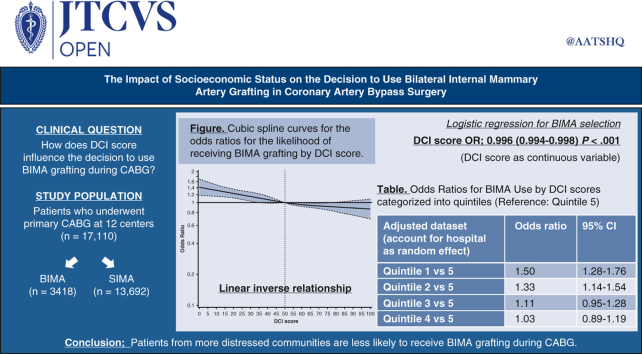

Lower likelihood of receiving bilateral internal mammary artery (BIMA) grafting among patients from more distressed communities.

**Figure:**
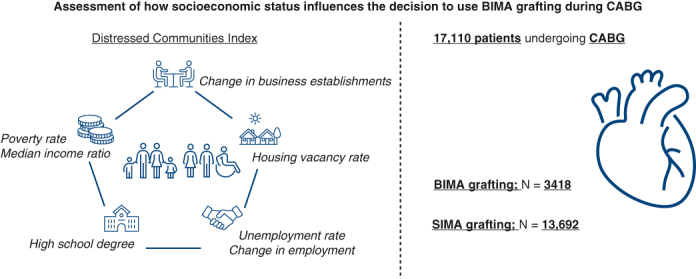
The influence of socioeconomic status on the decision to BIMA use in CABG.

**Central Message**

The Distressed Communities Index was independently associated with a lower likelihood of receiving BIMA grafting, but it was not associated with any major postoperative outcomes.

**Perspective**

Although graft selection plays a key role in improving outcomes after CABG, the influence of socioeconomic status on the choice between BIMA and SIMA grafting remains unclear. Our study shows that patients from more distressed communities are less likely to receive BIMA grafting. This suggests that surgeons may, perhaps unconsciously, factor in socioeconomic conditions when making grafting decisions.

Bilateral internal mammary artery (BIMA) grafting has been recommended for coronary artery bypass grafting (CABG) in appropriate patients, because several observational studies and meta-analyses reported the benefit of BIMA comparing single IMA (SIMA) in CABG.^1^^,^^2^ In clinical practice, average BIMA utilization was as low as 6.7% in isolated CABG across the United States.^3^ This discrepancy highlights a considerable lack of consensus regarding the most appropriate use of BIMA grafting. We therefore sought to explore the potential influence of socioeconomic factors in the decision-making process related to BIMA grafting.

Recently, the Distressed Communities Index (DCI) has emerged as a composite measure of community-level socioeconomic status, based on zip code.^4^ Several studies have examined the influence of the DCI on surgical outcomes.^4^, ^5^, ^6^, ^7^ Furthermore, it has been reported to be associated with decision making in the treatment of cardiac disease. Strobel and colleagues^8^ reported that increasing DCI is independently associated with odds of receiving percutaneous coronary intervention (PCI) over CABG. Thus, the DCI score was suggested to be an important factor influencing both decision making and surgical outcomes. Although graft selection is also a key factor in improving outcomes after CABG, no research to date has investigated how socioeconomic status influences the decision to use BIMA grafting during CABG and its subsequent influence on outcomes. In this study, we examine the influence of DCI on the decision-making process for BIMA grafting.

## Patients and Methods

### Study Design and Patient Selection

This study received approval from the Columbia University institutional review board (AAAT7723, May 2025 and AAAT7724, April 2025) with waiver of consent. The study population consisted of all patients who underwent CABG in any of the 12 cardiac surgery programs affiliated with the Columbia University HeartSource program from 2015 to 2024.^9^, ^10^, ^11^ Columbia HeartSource is an outreach project arising from the Divisions of Cardiac Surgery and Cardiology of Columbia University Medical Center that assists affiliate programs with quality oversight and program development in cardiovascular care.^10^ HeartSource is a diverse network, including both large and small programs, each with a distinct surgical team, in academic and nonacademic environments as well as urban, suburban, and rural settings across the United States (Table E1).^10^ The data used in the study were extracted from the Society of Thoracic Surgeons (STS) Adult Cardiac Surgery Database. Exclusion criteria included patients without an IMA graft (n = 552), with 1 distal anastomosis (n = 958). Additionally, 127 patients were excluded for having undergone previous CABG, which clearly precluded BIMA grafting. Twenty-one patients were excluded due to missing data on IMA grafting, and 1019 were excluded due to missing or incomplete zip code information. The zip code data contained in the STS database was used to link patients to the Economic Innovation Group's DCI. Postoperative outcomes—including reoperation, stroke, prolonged ventilation, renal failure, operative mortality, and deep sternal infection—were compared between patients who received BIMA grafting and those who received SIMA grafting. Renal failure was defined as acute renal failure or worsening renal function resulting in 1 or more of the following: a 3-fold increase in serum creatinine level, a serum creatinine level >4.0 mg/dL with an increase of at least 0.5 mg/dL, or the new onset of dialysis requirement postoperatively. Prolonged ventilation was defined as the need for mechanical ventilation for more than 24 hours after surgery.

### DCI

Use of the DCI in cardiac surgery has been described previously.^4^^,^^5^^,^^7^ The DCI is available for all zip codes with more than 500 residents, which captures 99% of the American population. The DCI produces a composite score of 7 metrics: percentage of residents with a high school degree, poverty rate, unemployment rate, housing vacancy rate, median income ratio, change in employment, and establishment. Each metric is evenly analyzed to produce a score for every zip code from 0 (no distress) to 100 (severe distress).

### Statistical Analysis

**Figure 1:**
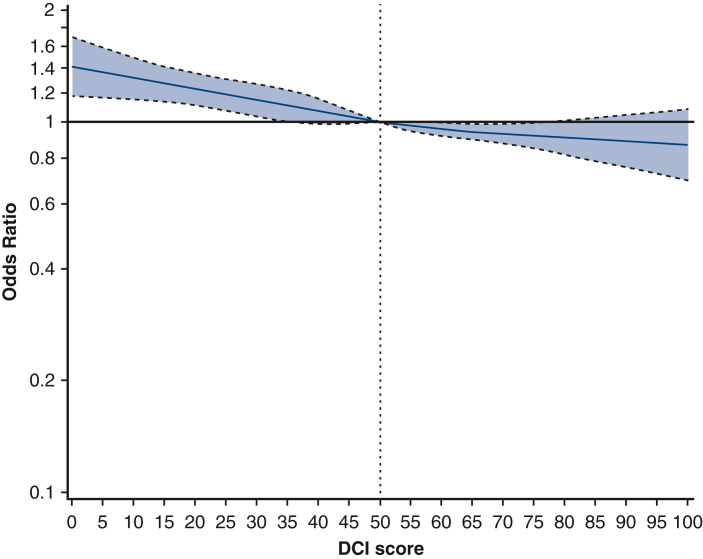
*Cubic spline curves* with 95% confidence bands for the odds ratios for the likelihood of receiving bilateral internal mammary artery grafting by Distressed Communities Index (DCI) score.

The cubic spline curve showed a relatively linear inverse relationship between DCI score and the odds ratio (OR) for the likelihood of receiving BIMA grafting with no specific cutoff point at which this association changes; therefore, DCI score was treated as a continuous variable (Figure 1). Continuous variables were expressed as mean ± SD or median (interquartile range) depending on normality, which was tested via the Shapiro-Wilk test, and were compared using Student *t*-test or Mann-Whitney *U* test, respectively. Categorical variables were expressed as numbers and percentages and were compared using χ^2^ test or Fisher exact test when appropriate. Missing data comprised a small proportion (<4.02%) and were imputed using the random forest algorithm (Table E2). Mixed effect logistic regression adjusting for hospital as a random effect was performed to identify independent factors associated with utilization of BIMA grafting. Variable selection for multivariable logistic regression model was partially informed by the results of univariable analysis with a *P* value < .10 but was ultimately determined by clinical acumen and prior literature to avoid overfitting. Furthermore, a mixed effect multivariable logistic regression analysis was performed to assess the association between BIMA grafting and DCI scores with postoperative complications, including mortality, stroke, renal failure, and prolonged ventilation, while adjusting for the current STS risk model, and adjusting for hospital as a random effect.^12^ No variables in the models were found to be highly correlated using the variance inflation factor (all variance inflation factors <5). We conducted a subgroup analysis to evaluate the association between the likelihood of receiving BIMA grafting and DCI scores categorized into quintiles. All statistical analyses were performed using SAS version 9.4 (SAS Institute Inc) and R version 4.4.1 (R Foundation for Statistical Computing).

## Result

### Patient Characteristics and Operative Details

**Figure 2:**
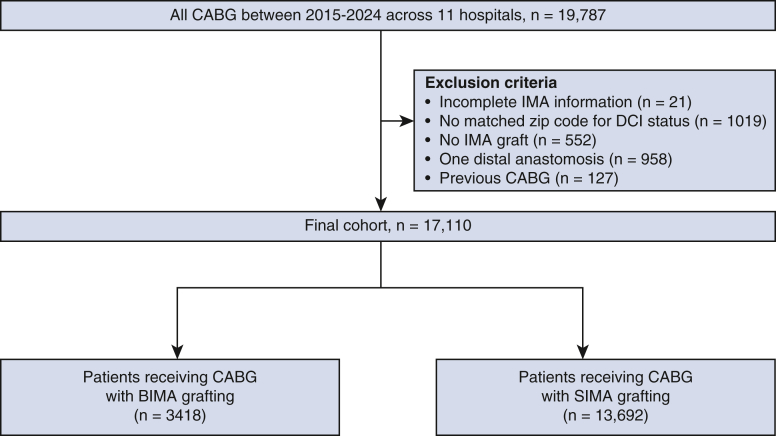
Consort diagram of patients undergoing coronary artery bypass grafting (CABG) with bilateral internal mammary artery (BIMA) or single internal mammary artery (SIMA). *DCI*, Distressed Communities Index.

**Figure E1:**
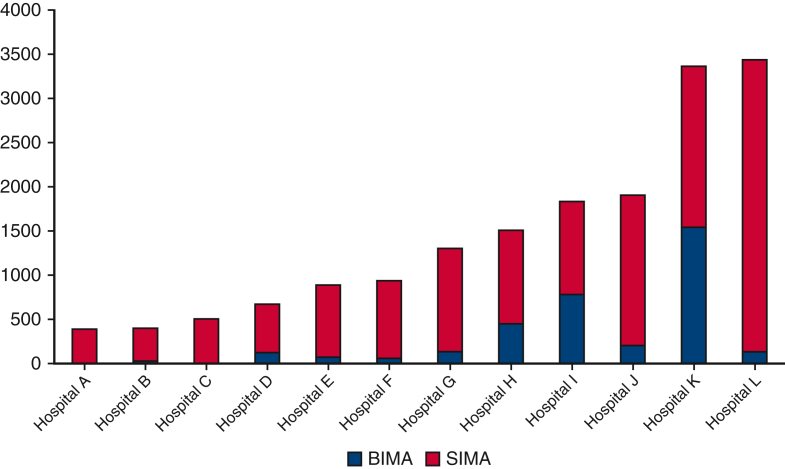
Distribution of bilateral internal mammary artery (BIMA) or single internal mammary artery (SIMA) by the center in the entire cohort.

A total of 17,110 patients who underwent CABG were included in the study (Figure 2). Of these patients, 13,692 patients (80.0%) received SIMA grafting, whereas 3418 patients (20.0%) received BIMA grafting. The proportion of patients who received BIMA grafting varied by center: more than 40% in 2 centers (hospitals I and K), 10% to 40% in 3 centers (hospitals D, H, and J), and <10% in 7 centers (Table E1 and Figure E1). The median age of the entire cohort was 67.0 years (range, 60.0-74.0 years) with significant difference between BIMA and SIMA groups (BIMA: 63.0; range, 56.0-70.0 years vs SIMA: 68.0 years; range, 61.0-74.0 years; *P* < .001), and BIMA was more frequently used in patients with fewer comorbidities than SIMA, except for heart failure, and previous PCI (Table 1).

**Table 1:** Patients’ characteristics and intraoperative outcomes

Variables	Total (N = 17,110)	BIMA (n = 3418)	SIMA (n = 13,692)	*P* value
Age (y)	67.0 (60.0-74.0)	63.0 (56.0-70.0)	68.0 (61.0-74.0)	<.0001
Female	3744 (21.9)	516 (15.1)	3228 (23.6)	<.0001
BMI	28.49 (25.38-32.27)	28.21 (25.41-31.7)	28.57 (25.35-32.42)	.002
Race				<.0001
White	13,825 (84.1)	2759 (85)	11,066 (83.9)	
Black	1206 (7.3)	207 (6.4)	999 (7.6)	
Asian	864 (5.3)	129 (4)	735 (5.6)	
Other	546 (3.3)	151 (4.7)	395 (3)	
Surgical era				.6771
Before 2020	7970 (46.6)	1603 (46.9)	6367 (46.5)	
After 2020	9140 (53.4)	1815 (53.1)	7325 (53.5)	
Preoperative comorbidity				
Diabetes	8450 (49.4)	1441 (42.2)	7009 (51.2)	<.0001
Hypertension	15,388 (90)	3005 (88)	12,383 (90.5)	<.0001
Liver disease	478 (2.8)	68 (2.0)	410 (3.0)	.001
Last creatinine level	1.0 (0.85-1.2)	1.0 (0.84-1.16)	1.0 (0.86-1.23)	<.0001
Dialysis	500 (2.9)	58 (1.7)	442 (3.2)	<.0001
Immunocompromise	702 (4.1)	98 (2.9)	604 (4.4)	<.0001
Chronic lung disease	4270 (25.0)	630 (18.4)	3640 (26.6)	<.0001
Peripheral arterial disease	2155 (12.6)	305 (8.9)	1850 (13.5)	<.0001
Cerebrovascular disease	3671 (21.5)	524 (15.4)	3147 (23)	<.0001
Previous myocardial infarction	8223 (48.3)	1415 (41.8)	6808 (49.9)	<.0001
Previous PCI	4805 (28.1)	923 (27)	3882 (28.4)	.1139
Heart failure	4910 (28.9)	974 (28.9)	3936 (28.9)	.9972
Cardiogenic shock	348 (2)	33 (1)	315 (2.3)	<.0001
LVEF	55 (45-60)	55 (48-60)	55 (45-60)	<.0001
DCI score	44.72 (23.45-68.62)	45.8 (24.24-70.63)	44.03 (23.39- 68.47)	<.001
Insurance				<.0001
Commercial	5824 (34)	1582 (46.3)	4242 (31)	
Medicare	8596 (50.2)	1277 (37.4)	7319 (53.5)	
Medicaid	1085 (6.3)	208 (6.1)	877 (6.4)	
Self and other	1604 (9.4)	351 (10.3)	1253 (9.2)	
Intraoperative data				
Status				<.0001
Elective	7095 (41.5)	1518 (44.4)	5577 (40.7)	
Urgent	9572 (55.9)	1862 (54.5)	7710 (56.3)	
Emergency/salvage	443 (2.6)	38 (1.1)	405 (3)	
Cardiopulmonary bypass time	85.0 (68.0-107.0)	85.0 (69.0-107.0)	85.0 (68.0-107.0)	.247
Crossclamp time	63.0 (50.0-78.0)	64.0 (50.0-81.0)	62.0 (50.0-78.0)	.001
Total number of anastomoses	3 (3-4)	4 (3-4)	3 (3-4)	<.0001
No. of anastomoses with arterial graft	1 (1-1)	2 (2-2)	1 (1, 1)	<.0001
No. of anastomoses with vein graft	2 (1-3)	1 (1-2)	2 (2-3)	<.0001
Radial artery use	888 (5.2)	135 (3.9)	753 (5.5)	.0003

values are presented as median (interquartile range) or n (%). *BIMA*, Bilateral internal mammary artery; *SIMA*, single internal mammary artery; *BMI*, body mass index; *PCI*, percutaneous coronary intervention; *LVEF*, left ventricular ejection fraction; *DCI*, Distressed Communities Index.

Operative characteristics were also shown in Table 1. BIMA group was more likely to have a higher number of total distal anastomoses (BIMA: 4.0; interquartile range [IQR], 3.0-4.0 vs SIMA: 3.0; IQR, 3.0-4.0; *P* < .001) and fewer anastomoses using saphenous vein graft (BIMA: 1.0; IQR, 1.0-2.0 vs SIMA: 2.0; IQR, 2.0-3.0; *P* < .001). Furthermore, the number of cases using radial artery was significantly lower in BIMA grafting (*P* < .001). There were no differences in cardiopulmonary bypass (*P* = .247), but aortic crossclamp time was longer in the BIMA group (*P* < .001).

### Association Between BIMA Grafting Use and DCI Score

The median DCI score is significantly different between BIMA (45.80; IQR, 24.29-70.63) and SIMA (44.03; IQR, 23.39-68.47) groups (*P* < .001) (Table 1). Multivariable logistic regression analysis accounting for site as a random effect showed that a higher DCI score was independently associated with a lower likelihood of receiving BIMA grafting (OR, 0.996; 95% CI, 0.994-0.998; *P* < .001), along with other preoperative factors such as age, sex, current smoking, diabetes, chronic lung disease, liver function, dialysis, immunosuppression, peripheral arterial disease, cerebrovascular disease, prior myocardial infarction, and left ventricular ejection fraction (all *P* values < .05) (Table 2). In subgroups analysis, when DCI score was divided by quintiles, negative association was observed between DCI score and the proportion of patients receiving BIMA grafting (Tables E3 and E4). However, after accounting for these wide differences in practice patterns, a higher DCI was actually associated with a lower likelihood of receiving BIMA grafting (Table E4).

**Table 2:** Logistic regression analysis for utilization of bilateral internal mammary artery (BIMA) grafting

Variables	Univariable analysis			Multivariable analysis		
	Odds ratio	95% CI	*P* value	Odds ratio	95% CI	*P* value
Age	0.95	0.95-0.95	<.001	0.94	0.93-0.94	<.001
Female	0.50	0.45-0.56	<.001	0.55	0.49-0.62	<.001
BMI	0.96	0.95-0.97	<.001	0.96	0.95-0.97	<.001
Race (reference category: White)						
Black	0.76	0.64-0.90	.0019	0.90	0.75-1.08	.274
Asian	1.05	0.83-1.32	.7003	0.85	0.67-1.08	.192
Other	0.88	0.71-1.09	.2332	0.67	0.53-0.84	.006
Surgical era						
Before 2020	0.69	0.64-0.76	<.001	0.68	0.62-0.75	<.001
Smoking (reference: No smoking)						
Current smoker	0.91	0.81-1.02	.1178	0.73	0.64-0.83	<.001
Previous smoker	0.79	0.72-0.87	<.001	0.93	0.84-1.03	.142
Diabetes	0.56	0.51-0.61	<.001	0.62	0.57-0.68	<.001
Hypertension	0.65	0.57-0.75	<.001	0.95	0.82-1.10	.496
Liver function	0.69	0.52-0.92	.011	0.74	0.55-0.99	.045
Dialysis	0.41	0.31-0.55	<.001	0.71	0.43-1.17	.179
Immunocompromise	0.68	0.54-0.86	.001	0.69	0.54-0.89	.004
Chronic lung disease (reference: None)	.		.			
Mild	0.72	0.64-0.82	<.001	0.88	0.77-1.00	.046
Moderate/severe	0.50	0.42-0.60	<.001	0.71	0.59-0.86	<.001
Peripheral arterial disease	0.59	0.51-0.67	<.001	0.84	0.72-0.97	.020
Cerebrovascular disease	0.58	0.52-0.65	<.001	0.79	0.70-0.89	<.001
Previous myocardial infarction	0.73	0.67-0.80	<.001	0.88	0.79-0.97	.014
Previous PCI	0.89	0.81-0.98	.015	0.99	0.89-1.10	.865
Heart failure	0.72	0.66-0.79	<.001	1.09	0.97-1.22	.142
LVEF	1.02	1.02-1.03	<.001	1.02	1.02-1.03	<.001
DCI score	0.995	0.993-0.996	<.001	0.996	0.994-0.998	<.001
Status (reference: Elective)						
Urgent	0.83	0.76-0.90	<.001	0.92	0.84-1.01	.082
Emergency	0.28	0.20-0.41	<.001	0.30	0.21-0.44	<.001

BMI, Body mass index; *PCI*, percutaneous coronary intervention; *LVEF*, left ventricular ejection fraction; *DCI*, Distressed Communities Index.

### Operative Outcome

In-hospital outcomes are presented in Table 3. Operative mortality lower among the BIMA patients (BIMA 1.2% vs SIMA 2.4%; *P* < .001), and the rate of renal failure (BIMA 1.1% vs SIMA 2.3%; *P* < .001) and prolonged ventilation (BIMA 5.7% vs SIMA 7.1%; *P* = .006) were also lower in BIMA groups, whereas the rate of deep sternal wound infection was higher in BIMA groups (BIMA 0.6% vs SIMA 0.2%; *P* = .0038) (Table 3). In the multivariable logistic regression analysis, the DCI score is not independently associated with any operative outcomes (all *P* values > .05), after adjusting for the STS risk estimates for each outcome (Table 4).

**Table 3:** Postoperative outcomes

Postoperative outcomes	Total (N = 17,110)	BIMA (n = 3418)	SIMA (n = 13,692)	*P* value
Reoperation	628 (3.7)	113 (3.3)	515 (3.8)	.2028
Stroke	199 (1.2)	30 (0.9)	169 (1.2)	.0812
Prolonged ventilation	1162 (6.8)	196 (5.7)	966 (7.1)	.0058
renal failure	355 (2.1)	36 (1.1)	319 (2.3)	<.0001
Combined mortality and morbidity	1835 (10.7)	313 (9.2)	1522 (11.1)	.0009
Operative mortality	366 (2.1)	40 (1.2)	326 (2.4)	<.0001
Deep sternal infection	53 (0.3)	19 (0.6)	34 (0.2)	.0038

Values are presented as n (%). *BIMA*, Bilateral internal mammary artery; *SIMA*, single internal mammary artery.

**Table 4:** Logistic regression analysis for postoperative outcomes

Variables	Odds ratio	95% CI	*P* value
Operative mortality			
DCI score	1.004	0.999-1.008	.0911
STS risk model	3.061	2.776-3.374	<.0001
BIMA	0.887	0.617-1.274	.5159
Renal failure			
DCI score	1.000	0.995-1.004	.9694
STS risk model	2.89	2.644-3.158	<.0001
BIMA	0.673	0.461-0.982	.0402
Deep sternal infection			
DCI score	1.008	0.997-1.019	.1723
STS risk model	2.142	1.512-3.036	<.0001
BIMA	1.782	0.964-3.296	.0654
Reoperation			
DCI score	1.003	1-1.006	0.0933
STS risk model	2.09	1.84-2.375	<.0001
BIMA	0.951	0.757-1.194	.663
Stroke			
DCI score	1.006	1-1.011	.0504
STS risk model	3.026	2.485-3.686	<.0001
BIMA	0.948	0.627-1.434	.8015
Prolonged ventilation			
DCI score	1.001	0.998-1.003	.5799
STS risk model	3.065	2.868-3.275	<.0001
BIMA	1.094	0.912-1.314	.333
Combined mortality and morbidity			
DCI score	1.002	1-1.004	.132
STS risk model	2.806	2.646-2.976	<.0001
BIMA	1.072	(0.924-1.242)	.3596

*DCI*, Distressed Communities Index; *STS*, Society of Thoracic Surgeons; *BIMA*, bilateral internal mammary artery.

## Discussion

The key implication of our study is that when accounting for the variability in practice among hospitals, higher DCI score (increased community distress) was independently associated with a lower likelihood of receiving BIMA grafting. BIMA grafting is currently recommended because it has been associated with reduced long-term mortality and fewer cardiac events compared with SIMA with saphenous vein grafting.^1^ Gaudino and colleagues^3^ previously reported that although BIMA grafting provides comparable long-term outcomes and similar operative mortality to the use of radial artery grafts when used as the second conduit, it is superior to saphenous vein grafting. However, in clinical practice, the use of BIMA grafting has not increased dramatically, and its frequency during CABG remains low. Iribarne and colleagues^13^ highlighted significant regional variation in BIMA use within the United States. Although they identified differences in institutional volume as 1 reason for this variation, we believe that socioeconomic status could be another contributing factor for decision making of BIMA use because it also varies regionally.^14^ Although Patrick and colleagues^15^ previously reported an association between lower socioeconomic status and the use of fewer arterial conduits during CABG, they did not specify the type of arterial graft used, and no studies have specifically examined the relationship between socioeconomic status and BIMA use. Therefore, we believe this study provides valuable insights into how the DCI score influences the decision to use BIMA grafts and its influence on patient outcomes.

In this study, when accounting for practice variability, higher DCI score was associated with the lower likelihood of receiving BIMA grafting. Previously, Strobel and colleagues^8^ suggested that poor financial tolerance contributes to the preference for PCI over CABG in high-DCI communities. Furthermore, Philbin and colleagues^16^ reported that lower income was a negative predictor of coronary revascularization, including CABG and PCI, among patients with acute myocardial infarction. Thus, insufficient financial or social resources have been reported to be associated with the determination of the indication for cardiac surgery. Among the major concerns with CABG using BIMA grafting is the increased risk of deep sternal wound infection, which can lead to prolonged hospitalization.^17^, ^18^, ^19^ Previously, Gray and colleagues^20^ reported that the mean costs were approximately 9% higher in patients undergoing BIMA grafting compared with those receiving SIMA grafting, primarily due to extended in-hospital stays and increased costs associated with sternal wound complications during follow-up. Furthermore, our findings are consistent with a previous study, which suggested that lower socioeconomic status was associated with the use of fewer arterial grafts during CABG, possibly due to a lack of strong primary care or cardiology advocates in lower socioeconomic populations.^15^ Therefore, we believe that the DCI score is also an important factor in graft selection because it serves as a more robust indicator of patient-level socioeconomic status.

In observational studies, there is concern that unmeasured selection bias may influence the observed findings, especially when interpreting the more favorable outcomes associated with CABG using BIMA grafting. Gaudino and colleagues^21^ pointed out that surgeons tend to perform CABG with BIMA grafting in patients perceived as healthier, with longer life expectancy from both a cardiac and general health perspective, which creates an unmeasured confounder that is difficult to quantify in observational studies. Socioeconomic factors are also known to have a major effect on quality of life and life expectancy.^5^^,^^22^^,^^23^ Thus, although several other unmeasured confounders may exist—such as frailty—our findings suggest that, in addition to financial resources, surgeons may unknowingly take socioeconomic factors into account as part of the eyeball test when deciding on BIMA grafting, due to their perceived impact on quality of life and life expectancy.

Several studies have demonstrated that DCI independently predicts risk-adjusted operative mortality after CABG.^4^^,^^5^ Mehaffey and colleagues^5^ and Strobel and colleagues^24^ reported that the DCI remained significantly associated with mortality (OR, 1.03; 95% CI, 1.02-1.04; *P* < .0001), even after adjusting for the STS-predicted risks for these outcomes. As a result, they suggested the potential utility of DCI as a risk-adjustment tool in cardiac surgery populations and the integration of the DCI score into the STS risk model.^5^ In the present study, whereas the current STS risk model shows a robust association with postoperative outcomes, DCI is not independently associated with operative outcomes. This may be due to the limited statistical power resulting from the small sample size. However, notably, the evaluation of the benefits of BIMA grafting over SIMA grafting focused primarily on long-term outcomes rather than short-term results. Therefore, long-term follow-up is warranted to determine whether DCI should be considered a confounding variable when comparing outcomes between BIMA and SIMA grafting.

### Study Limitations

We acknowledge the limitations of the present study. First, the retrospective design may introduce unmeasured confounding variables. Second, the small sample size limited the statistical power of our analysis. Third, 5.6% of the population was excluded for missing zip code, which may introduce selection bias into this retrospective study. Fourth, because the goal of this study was to examine the influence of socioeconomic status on the use of BIMA grafting rather than SIMA grafting, cases in which the radial artery was used as the second arterial graft in addition to IMA grafting were also included. The number of such cases differed significantly between groups. Fifth, because postoperative medical management, particularly wound care and medication use, was not uniformly collected, these factors were not included in the study. Therefore, their potential influence on outcome differences remains unknown. Sixth, information on harvesting approaches and grafting patterns was not uniformly collected and was therefore excluded from this study, although these factors have been reported to be associated with postoperative outcomes, such as deep wound infections.^25^ Moreover, surgical experience is known to play a critical role in determining outcomes after CABG.^26^^,^^27^ Accordingly, we acknowledge that the absence of data on surgeon experience limits the generalizability of our findings. Seventh, as previously discussed, we acknowledge that several other unmeasured confounders may exist. Therefore, although our findings suggest that the DCI should be recognized as a potential confounder when comparing outcomes between BIMA and SIMA grafting, the possibility of selection bias remains a concern. Finally, we lack data on long-term outcomes. As noted above, long-term follow-up is essential when comparing outcomes between BIMA and SIMA grafting. However, our findings suggest that surgeons may consider socioeconomic factors when deciding on BIMA grafting. This indicates that DCI should be recognized as a potential confounder in observational studies.

## Conclusions

Patients from more distressed communities are less likely to receive BIMA grafting. This suggests that surgeons may unknowingly consider socioeconomic factors as part of their decision making for BIMA grafting. Although the DCI score did not significantly influence operative outcomes after adjusting for the variables included in the STS risk estimate, long-term follow-up is warranted to determine whether DCI should be considered a confounding variable when comparing outcomes between BIMA and SIMA grafting.

## Conflict of Interest Statement

The authors reported no conflicts of interest.

The *Journal* policy requires editors and reviewers to disclose conflicts of interest and to decline handling or reviewing manuscripts for which they may have a conflict of interest. The editors and reviewers of this article have no conflicts of interest.

## Figures and Tables

**Table E1:** Hospital characteristics and bilateral internal mammary artery (BIMA) use

Hospital	Major/minor urban/rural∗	Academic†	Approximate annual volume‡	State	Median DCI	BIMA use (%)
A	Major urban	Yes	130	New York	55	1.3
B	Minor urban	No	90	Connecticut	50	3.5
C	Major urban	Yes	165	New York	62	1.8
D	Rural	No	120	New York	71	16.6
E	Major urban	No	500	Tennessee	53	6.9
F	Major urban	No	205	New York	44	4.8
G	Major urban	Yes	795	New York	45	9.3
H	Major urban	No	360	Florida	44	29.1
I	Major urban	No	435	Tennessee	68	42.1
J	Minor urban	No	275	South Carolina	41	10.2
K	Major urban	Yes	1005	New York	49	45.4
L	Minor Urban	No	950	Florida	27	3.5

*DCI*, Distressed Communities Index.

∗ Minor urban indicates the population of the city where hospital is located is < 1,50,000, major urban indicates the population of the city where the hospital located is > 150,000, and rural indicates the city where the hospital is located is in a rural setting.

† Academic uses Society of Thoracic Surgeons definition as center with a cardiac surgery teaching program.

‡ Approximate annual volume of all on-pump nontransplant/nonventricular assist device cardiac surgery operations.

**Table E2:** Missing data

Variable	Missing data
BMI	2 (0.01)
Race	669 (3.91)
Hispanic	688 (4.02)
Diabetes	12 (0.07)
Hypertension	10 (0.06)
Liver disease	25 (0.15)
Dialysis	4 (0.02)
Immunocompromise	10 (0.06)
Chronic lung disease	649 (3.79)
Peripheral arterial disease	28 (0.16)
Cerebrovascular disease	25 (0.15)
Previous myocardial infarction	73 (0.43)
Previous PCI	10 (0.06)
Previous valve procedures	10 (0.06)
Heart failure	118 (0.69)
LVEF	235 (1.37)
Cardiopulmonary bypass time	210 (1.23)
Crossclamp time	390 (2.28)

Values are presented as n (%). *BMI*, Body mass index; *PCI*, percutaneous coronary intervention; *LVEF*, left ventricular ejection fraction.

**Table E3:** Proportion of patients receiving bilateral internal thoracic artery (BIMA) grafting across Distressed Communities Index (DCI) score quintiles

DCI score quintile	Total (N = 17,110)	BIMA N = 3418	SIMA N = 13,692	*P* value	BIMA use (%)
DCI score				<.0001	
Quintile 1 (lowest distress)	2991 (17.5)	602 (17.6)	2389 (17.4)		20.13
Quintile 2	4361 (25.5)	830 (24.3)	3531 (25.8)		19.03
Quintile 3	3990 (23.3)	698 (20.4)	3292 (24)		17.49
Quintile 4	3744 (21.9)	825 (24.1)	2919 (21.3)		22.04
Quintile 5 (highest distress)	2024 (11.8)	463 (13.5)	1561 (11.4)		22.88

Values are presented as n(%). *SIMA*, Single internal thoracic artery.

**Table E4:** Odds ratios for bilateral internal thoracic artery (BIMA) use by Distressed Communities Index (DCI) quintiles (reference category: Quintile 5)

Comparison (Quintile vs 5)	Odds ratio	95% CI
Unadjusted dataset		
Quintile 1 vs 5	0.85	0.74-0.97
Quintile 2 vs 5	0.79	0.70-0.90
Quintile 3 vs 5	0.72	0.63-0.82
Quintile 4 vs 5	0.95	0.84-1.08
Adjusted dataset∗		
Quintile 1 vs 5	1.50	1.28-1.76
Quintile 2 vs 5	1.33	1.14-1.54
Quintile 3 vs 5	1.11	0.95-1.28
Quintile 4 vs 5	1.03	0.89-1.19

∗ Accounts for hospital as random effect.
